# Atlanta Academy of Medicine

**Published:** 1878-04

**Authors:** J. G. Westmoreland

**Affiliations:** Reporter


### Reports of Societies.

### ATLANTA ACADEMY OF MEDICINE.

J. G. WESTMORELAND, M.D., Reporter.

Atlanta, Ga., March 4, 1878.

Dr. J. F. Alexander, President, in the chair.

Dr. H. L. Wilson reported a case of sudden death under the following circumstances: A lady 52 years old was well at supper, and, as was her custom, reclined upon her bed after tea. Suddenly she exhibited strange appearance in the eyes, and was found to be unconscions, pulseless, and died at once. He thought embolism of the brain a probable cause, and desired the opinion of members on the subject.

Dr. Baird thought the symptoms were those most likely to result from rupture of a blood vessel in the brain, probably in the neighborhood of the medulla oblongata.

Dr. L. G. Alexander differed from those preceding him as to the cause of death in the case reported. From his discoveries by several autopsies in cases of death with similar symptoms, he was decidedly of the opinion that the cause existed in rupture of the heart from fatty degeneration.

Dr. H. L. Wilson reported also a case of stricture of the urethra and of the meatus urinarius, in which relief was afforded at once by enlarging the canal with ceviales urethrotome and the meatus by the meatretome. The permanent stricture requiring the cutting instrument was situated about two inches from the meatus, and at the bulb of the urethra spasmodic constriction existed, requiring dilation by the bougie only. He is of opinion both the spasmodic and permanent stricture are the result of contracted meatus.

Dr. W. F. Westmoreland presented a specimen of congenital deformity of the middle and index fingers, requiring amputation of the former at the metacarpal articulation with a portion of the metacarpal bone and two falanges of the index finger. The portion amputated of the middle finger measures about two inches in diameter and nine inches long. The patient from which the specimens were obtained was a man about thirty years old, and in other respects perfect. These fingers were proportionately large at birth.

---

Atlanta, Ga., March 11, 1878.

Dr. J. F. Alexander, President, in the chair.

Dr. King reported a case of a female 28 years old with enlargement of the abdomen, for five months commencing—as reported—by a small tumor, in left illiac fossa. He was called December 5, 1877, and found her suffering with pain similar to the throes of labor. The patient was feeble and gave evidences of speedy dissolution. Opiates were given to allay pain and give rest. The diagnosis formed at the time was that an enlargement existed in the ovary, or tumor in the broad ligament. Consultation was had, and the tumor pierced with an aspirator, through the left lateral wall of the vagina, and about a pint of thick straw-colored fluid escaped. The patient was then directed to take regularly a saturated solution of chlorate of potassium, with iron and bitter tonics, more with the view of affording a placebo than of effecting a cure of the case. Strange to say, however, improvement commenced and steadily progressed. Instead of increase of the enlargement, she gradually became smaller as her general health improved, until reduced to the normal size. Menstruation occurred two weeks since, for the first time since commencement of the enlargement, four or five months since. Dr. K. is unable to determine whether to the chlorate or the tapping was due the cure, which is certainly remarkable, under all the circumstances of the case.

Dr. W. F. Westmoreland presented a specimen of malformation of the thumb in a girl eight years old. The case exhibited a double thumb, the bifercation occurring at the articulation of the first phalanx with the metacarpal bone. The two were of about equal size, and in this particular differed from all the cases of this kind of deformity he had before seen. The operation consisted of amputating the bone, with the soft parts, of the external thumb.

He also asked of members whether they had seen any influence in the way of relief from whooping cough follow vaccination, or used it as a prophylactic.

Dr. J. F. Alexander, the President, stated that at one time during the prevalence of whooping cough vaccination had in families where the disease existed was found to moderate the symptoms, and his attention being called to the fact thus accidentally discovered, he has advised and practiced vaccination, with the view of lessening the violence of the cough.

Adjourned.

